# Economic costs of alcohol use in Sri Lanka

**DOI:** 10.1371/journal.pone.0198640

**Published:** 2018-06-07

**Authors:** Sajeeva Ranaweera, Hemantha Amarasinghe, Nadeeka Chandraratne, Montarat Thavorncharoensap, Thushara Ranasinghe, Sumudu Karunaratna, Dinesh Kumara, Benjarin Santatiwongchai, Usa Chaikledkaew, Palitha Abeykoon, Amala De Silva

**Affiliations:** 1 Sri Lanka Medical Association, Colombo, Sri Lanka; 2 Ministry of Health, Nutrition and Indigenous Medicine, Institute of Oral Health, Maharagama, Sri Lanka; 3 Department of Pharmacy, Faculty of Pharmacy, Mahidol University, Bangkok, Thailand; 4 World Health Organization Country Office, Colombo, Sri Lanka; 5 Ministry of Health, Nutrition and Indigenous Medicine, Colombo, Sri Lanka; 6 Ministry of Health, Nutrition and Indigenous Medicine, National Authority on Tobacco and Alcohol, Battaramulla, Sri Lanka; 7 Health Intervention and Technology Assessment Program, Ministry of Public Health, Nonthaburi, Thailand; 8 University of Colombo, Colombo, Sri Lanka; Edith Cowan University, AUSTRALIA

## Abstract

**Aim:**

Alcohol related disease conditions are responsible for a significant proportion of morbidity and mortality in Sri Lanka. This study quantified the economic cost of selected alcohol related disease conditions in Sri Lanka in 2015.

**Methods:**

This study uses the prevalence-based cost of illness methodology specified by the World Health Organization, and uses the gross costing approach. The direct costs includes the costs of curative care (inpatient and outpatient care borne by the state and out of pocket expenditure borne by patients) for alcohol related diseases, weighted by the respective population attributable fractions. Indirect costs consist of lost earnings due to absenteeism of the patient and carers due to seeking care and recuperation, and the loss of income due to mortality.

Data form the Ministry of Health, Registrar General’s Department, Department of Census and Statistics and the National Cancer Registry was used. Systemic and house costs and population attributable fractions were obtained from research studies. Economists, Public Health Experts, Medical Administrators and Clinical Specialists were iteratively consulted during the estimation and validation of the costs and the results.

**Results:**

The estimated present value of current and future economic cost of the alcohol-related conditions for Sri Lanka in 2015 was USD 885.86 million, 1.07% of the GDP of that year. The direct cost of alcohol related disease conditions was USD 388.35 million, which was 44% of the total cost, while the indirect cost was USD 497.50 million, which was 66% of the total cost. Road Injury cost was the highest cost category among the conditions studied.

**Conclusion:**

Addressing alcohol use and its harms through effective implementation of evidence-based polices and interventions is urgently required to address the economic costs of alcohol use in Sri Lanka as it imposes a significant burden to the country.

## Introduction

Alcohol causes a wide variety of diseases imposing a substantial social and economic burden on societies. Globally, alcohol is responsible for approximately 3.3 million deaths annually which is 5.9% of all deaths [1].

Alcohol use is related to adverse medical conditions, injuries, accidents, violence and crime [2] [3] [4] [5]. Alcohol harms humans through several mechanisms. Acetaldehyde, the main metabolic product of alcohol is a potent human carcinogen which causes cancer by damaging DNA and stopping our cells from repairing these damages [6]. Various biological mechanisms have also been identified for causation of cardiovascular disorders which include cardiomyopathy, hypertension, coronary artery diseases and stroke [7].

Alcohol-use disorders, especially among men, are the most disabling disease categories in the global burden of disease [8]. More than 200 ICD-10 three-digit disease codes exist in which alcohol is part or a component cause and more than 70 other ICD-10 three-digit or four-digit codes include alcohol in their name or definition [8], indicating that alcohol consumption is a necessary cause.

The negative consequences of alcohol on people other than the drinker include injuries and deaths from road traffic accidents, harm from interpersonal violence, aggression and crime, harm to families that include psychological distress, pain and suffering from domestic violence, marital separation and divorce, child and household neglect, poverty, and, harm to the developing foetus [9] [10].

The economic consequences of expenditure on alcohol can be significant at household level. Besides money spent on alcohol, a heavy drinker also faces other adverse economic effects. These include low wages (because of missed work and reduced efficiency on the job), lost employment opportunities, increased medical expenses for illness and accidents, legal cost of drink-related offences, and decreased eligibility for loans [11]. The opportunity cost of expenditure on alcohol is most severe for the lower income category as well [12]. The negative economic consequences on households, exerts a substantial burden on the national economy [9].

Apart from an unhealthy population with reduced productivity hindering the development of the country, a considerable proportion of national health expenditure has to be spent to treat alcohol related diseases. Systemic reviews show that the economic cost of alcohol ranged from 0.45% to 5.44% of each country’s Gross Domestic Product (GDP) [13]. For high-income and middle-income countries, the economic cost of alcohol was estimated to be greater than 1% of the Gross National Product (GNP) [4]. In most countries, indirect costs due to premature mortality, disability, and absenteeism was considered as the largest part of the total costs [13]. A recent systematic review conducted in 19 European countries indicated that the social cost of alcohol per-capita ranged from €26 to €1500 [14].

Sri Lanka is a middle-income country with a population of 21 million and a GDP per-capita of USD 3900 [15]. The World Health Organization (WHO) STEPWise approach to chronic disease risk factor survey in Sri Lanka in 2015, showed that 34.8% males and 0.5% females consumed alcohol during last 30 days [16]. This study also showed that the prevalence of heavy drinking was 17% among males. The Sri Lanka National Youth Survey in 2012 reported nearly 10% of males between the ages of 15–19 years had consumed alcohol during the past week [17]. A study in an urban population reported the prevalence of lifetime alcohol abuse and dependence as 6.2% and 4% respectively [18]. Prevalence of drinking among males has shown to be significantly higher compared to females, [16] [18] [19]. Studies have also shown a higher prevalence of alcohol consumption among males in urban than in rural population [18] [20].

There is a paucity of published studies on the economic impact of alcohol and its related conditions in Sri Lanka, although there have been recent publications of social costs of alcohol use such as poverty [11] [12] This study was conducted by the National Authority on Tobacco and Alcohol, Sri Lanka Medical Association, Country office of the World Health Organization (WHO), and Health Intervention and Technology Assessment Programme, Thailand as part of the WHO SEARO initiative on introducing and capacity building on Health technology Assessments among South East Asian countries. The objective of the study was to estimate the economic costs of alcohol in Sri Lanka for the year 2015.

## Methodology

The prevalence-based gross costing approach was adopted for calculation of the costs. The direct economic costs of alcohol related diseases consisted of costs to treat alcohol related disorders in the state sector hospitals in 2015 which include the costs borne by the government and the out-of-pocket (OOPE) expenditures borne by the patients for outpatient and inpatient visits and the follow-up clinic visits during 2015. The indirect cost calculated consisted of the costs of productivity loss due to mortality and absenteeism from work. The calculations were made in local currency and converted to United States Dollars (USD) at the 2015 mid-year exchange rate of 135 Sri Lanka Rupees per USD [15].

### Calculation of alcohol attributable fractions (AF)

The following formula is used [21]:

$$AF=\frac{\sum_{j=1}^{n}P_{j}({RR}_{j}-1)}{\sum_{j=1}^{n}P_{j}({RR}_{j}-1)+1}$$

Exposure category is j. The baseline exposure or no exposure (j = 0), RR (j) is taken as the relative risk at exposure level j compared with no consumption. P (j) is the prevalence of the j^th^ category of exposure.

The number of deaths and health care episodes is used to estimate the number of health care episodes attributable to alcohol by applying the Attributable Fraction derived from this formula, where directly applicable AAF relevant to Sri Lanka was not available in the literature.

### Main cost components

The government expenditure and out-of-pocket (OOPE) expenditures for outpatient and inpatient visits as well as clinic visits consisted of direct health care costs. The frequency of clinic visits per year and the cost borne by the government providing such services for each person were taken as the direct costs for outpatient care. The direct inpatient care costs were calculated using the scenario building approach using expert panels and published data. Two different expert panels were convened, which is discussed below, to estimate the direct costs of cancers and for non-cancer conditions related to alcohol use.

Indirect costs consisted of both costs of premature mortality and the costs of absenteeism. The economic costs of premature mortality was estimated using the human capital approach. In this approach, lost value of a worker’s production is estimated using present earnings plus a discounted rate of future earnings. Therefore, this calculation takes in to account the costs of loss of life or withdrawal from the workforce, taking into account and the earnings for the period up to retirement. In this study, this cost was estimated using the average earnings, the probabilities of survival and employment, and discounting to the future. The daily earnings based on average earnings was used to measure absenteeism.

### Use of expert groups

Three different panel of experts convened through a nominal group technique aided in consensus generation and validation of the study. The first two groups were to estimate and validate the treatment costs and the third to validate the overall methodology and the results. The first group consisted of experts in public health, senior consultant oncologists, consultant oncological surgeons, medical administrators and nursing staff who estimated the treatment costs related to alcohol related cancers. The second expert group consisted of public health experts, senior economists and clinical consultants in surgery, general medicine (internal medicine) and psychiatry. This group worked on alcohol related conditions other than cancer.

These two expert groups estimated and validated through consensus the following for each alcohol related condition: the average age of onset, the duration of hospital stay, requirement surgery and days of intensive care, costs of surgery and pharmaceutical drugs and devices and the average number of clinic visits. Where there was published data available on these variables, it was taken into consideration by both the panels during the estimation process. This data was used for the calculation of the direct treatment costs, out of pocket expenditure (OOPE), and the loss of productivity due to the disease conditions.

The final validation of the findings of the study was through an expert group that consisted of experts in economics, research methodology, public health and consultants in different disciplines of clinical medicine and surgery that deliberated the data used, the methods of analysis and the final results of the study.

### Data sources

Cancer incidence data for males and females are projected for 2015 based on data from National Cancer Registry 2009 [22] and compared with Globocan 2012 IARC data base for Sri Lanka [23]. Hospital admissions, deaths and clinic visit data on other conditions for 2015 was obtained from the indoor morbidity and mortality data published in the Annual Health Bulletin 2015, Ministry of Health [24].

The other sources of information were Department of Census and Statistics (income) and published reports of surveys conducted by government and non-governmental organizations. All the above sources of data had a coverage of 100%, with data available from all parts of the country. Where there was a lack of robust data, data from postgraduate studies undertaken on community medicine and health economics were used.

The extracted morbidity, mortality and cost data were tabulated, reviewed and validated through an iterative process of meetings of experts, which was discussed above. The final decisions on the validity, quality and the robustness of the extracted data and the technical strength of the methodology for costing different outcomes were reached through the consensus of the experts.

The completeness and timeliness of the reports published data from different government department and ministries were not uniform in some instances. This resulted a need for estimating the variable of interest based on the latest available data. However, such estimations were discussed with the panel of experts as stated above, before being used for the analysis.

### Calculation of direct costs

The Centre for Disease Control and Prevention (CDC) identifies 54 acute and chronic disease conditions attributable to alcohol [25]. Due to limitations in the availability of data, 8 types of cancers and 19 noncommunicable diseases (NCD) were considered for the present study. Alcohol Attributable Fractions (AF) for each selected condition was sought by a comprehensive literature survey. Tables 1 and 2 present the conditions that were considered for morbidity and mortality cost calculations along with the attributable fractions used for each condition. The AFs together with the number of deaths and health care episodes were used to estimate the mortality and morbidity attributable to alcohol use in this study.

**Table 1:** Attributable fractions used for morbidity due to alcohol.

**Cancers**				
**Site**	**AF**		**ICD O-3 Revision**	**Source of RR/AF**
	**Male**	**Female**		
Breast	18.37%	1.44%	C 50	Bagnardi et al. 2015 [26]
Colorectal	13.97%	1.04%	C 18–21	Bagnardi et al. 2015 [26]
Larynx	37.84%	3.81%	C 32	Bagnardi et al. 2015 [26]
Lip, oral, cavity, pharynx	61.01%	9.24%	C00- C14 except C7-8	Tramacere, et al. 2010 [27]
Liver	28.31%	2.50%	C 22	Bagnardi et al. 2015 [26]
Oesophagus	59.31%	8.66%	C 15	Bagnardi et al. 2015 [26]
Pancreas	6.55%	0.45%	C 25	Bagnardi et al. 2015 [26]
Stomach	7.19%	0.50%	C 16	Bagnardi et al. 2015 [26]
**Noncommunicable diseases and other conditions**				
**Condition**	**AF**		**ICD 10**	**Source of RR/AF**
	**Male**	**Female**		
Acute and chronic pancreatitis	21.00%	3.60%	K85, K86.1	IHME, 2013 [28]
Alcohol use disorders	100.00%	100.00%	F10.3-F10.9, F10.0, F10.1, F10.2, G62.1, G31.2, G72.1, I42.6, K29.2, K70-K70.4, K70.9	IHME,2013 [28]
Alcoholic Gastritis and Duodenitis	100.0%	100.0%	K29	IHME, 2013 [28]
Alcoholic liver disease	100.00%	100.00%	K70-K70.4, K70.9	IHME, 2013 [28]
Cerebrovascular disease	5.72%	0.37%	I60—I69	IHME, 2013 [28]
Cholethaiasis & cholecystitis	18%	—	K80,K81	IHME, 2013 [28]
Diabetes mellitus	1.01%	0.07%	E 11	IHME, 2013 [28]
Epilepsy	5.19%	0.45%	G40, G41	IHME, 2013 [28]
Fire injuries/ burning	1.79%	0.09%	X00-X09	IHME, 2013 [28]
Hypertension	3.13%	0.12%	I10-I15	IHME,2013 [28]
Ischemic Heart Disease	8.00%	8.00%	I20-I25	Leong et al, 2014 [29]
LRTI	2.92%	0.46%	J12-J18	IHME,2013 [28]
Other diseases of liver	20.36%	6.45%	K71-K76	IHME, 2013 [28]
Poisoning	20.00%	20.00%	X40-X49 [except X45]	Expert Opinion
Road Injuries	32.00%	32.00%	V 01-V89, Y 85	Zeisser et al., 2013 [30]
Self-harm	27.70%	17.20%	X60-X84, [except X65] Y87.0	Single et al, 2000 [31]
Supra Ventricular Cardiac Arrhythmia	8.50%	2.19%	I47.1, I47.9, I48	IHME, 2013 [28]
Tuberculosis	5.86%	0.56%	A15-A16	IHME,2013 [28]
Undiagnosed / Uncoded [c]	20.0%	5.0%	Uncoded	Expert Opinion

**Table 2:** Alcohol attributable fractions used for mortality due to alcohol.

**Cancers**				
**Site**	**AF**		**ICD O-3 Revision**	**Source of RR/AF**
	**Male**	**Female**		
Breast	18.4%	1.4%	C 18–21	IHME, 2013 [28]
Colorectal	14.0%	1.0%	C 18–21	IHME, 2013 [28]
Larynx	37.8%	3.8%	C 32	IHME, 2013 [28]
Lip, oral, cavity, pharynx	61.0%	9.2%	C00- C14 except C7-8	IHME, 2013 [28]
Liver	28.3%	2.5%	C 22	IHME, 2013 [28]
Oesophagus	59.3%	8.7%	C 15	IHME, 2013 [28]
Pancreas	6.6%	0.5%	C 25	IHME, 2013 [28]
Stomach	7.2%	0.5%	C 16	IHME, 2013 [28]
**Noncommunicable Diseases and other conditions**				
**Condition**	**AF**		**ICD 10**	**Source of RR/AF**
	**Male**	**Female**		
Acute and chronic pancreatitis	22.0%	0.0%	K85, K86.1	IHME,2013 [28]
Alcohol use disorders	100.00%	100.00%	F10.3-F10.9, F10.0, F10.1, F10.2, G62.1, G31.2, G72.1, I42.6, K29.2, K70-K70.4, K70.9	IHME,2013 [28]
Alcoholic Gastritis and Duodenitis	100.0%	100.0%	K29	Rehm et al., 2009 [4]
Alcoholic Liver Disease	100.0%	100.0%	K74.69	Rehm et al., 2009 [4]
Cerebrovascular disease	5.0%	0.0%	I60—I69	Rehm et al., 2009 [4]
Cholethaiasis & cholecystitis	18.0%	0.0%	K80,K81	Rehm et al., 2009 [4]
Diabetes mellitus	1%	1%	E10—E14	IHME,2013 [28]
Drowning	42.0%	41.0%	W65-W74	Rehm et al., 2009 [4]
Epilepsy	5.3%	0.35%	G40, G41	Rehm et al., 2009 [4]
Falls	7%	7%	WOO- W19	IHME,2013 [28]
Fire injuries/ burning	21.0%	10.0%	X00-X09	Rehm et al., 2009 [4]
Homicide	7.2%	7.2%	X85-Y09	IHME,2013 [28]
Hypertension	3.1%	0.1%	I10-I15	IHME,2013 [28]
Ischemic Heart Disease	8.0%	8.0%	I20-I25	Leong et al, 2014 [29]
LRTI	54.0%	31.0%	J12-J18	IHME,2013 [28]
Other diseases of liver	20.36%	6.5%	K71-K76	IHME,2013 [28]
Poisoning	22.0%	20.0%	X40-X49 (except X45)	Rehm et al., 2009 [4]
Road Injuries	53.0%	25.0%	V 01-V89, Y 85	Rehm et al., 2009 [4]
Self-harm	27.0%	17.0%	X60-X84, (except X65) Y87.0	Single et al., 2000 [31]
Supra Ventricular Cardiac Arrhythmia	31.0%	20.0%	I47.1, I47.9, I48	Rehm et al., 2009 [4]
Tuberculosis	5.9%	0.6%	A15-A16	IHME,2013 [28]
Undiagnosed/Uncoded (c)	10.0%	5.0%	Uncoded	Expert Opinion

The undiagnosed / uncoded morbidity and mortality of all conditions in Sri Lanka consisted of a sizable number. Therefore, not considering these would have significantly underestimated the cost. However, despite the many conditions related to alcohol that could have been within these cases, the expert opinion was to use conservative AFs of 0.2 for morbidity and 0.1 for mortality of males and 0.1 and 0.05 for the morbidity and mortality of females.

The costs of inpatient care took into account the hotel costs and the costs of pharmaceuticals, investigations, surgery and the costs of intensive care specific for each disease condition. These costs were iteratively refined by the panel of experts, who had experience of working in both the government and the private health sectors. The OOPE consists of the costs borne by the family of the patent during the hospital admission and clinic visits.

### Calculation of indirect costs

Both morbidity and mortality were considered for calculating the indirect costs. The “Scenario Building Method” was used for calculating the costs of premature mortality. This was based on the following: average income as reported in the Sri Lanka National Household income and Expenditure Survey 2012/2013 (HIES), annual economic growth, weights for probability of survival and employment with the volume of lost earnings depending on age of death and assumed age of retirement. A discount rate of 4.5% was used to convert the future values to the present value.

The absenteeism cost was the lost income of the patients and the carers due to treatment seeking, hospitalization and recuperation at home following hospitalization. It incorporates the average monthly earnings as reported in Sri Lanka HIES, extrapolated for 2015 based on inflation indexing.

## Results

### Direct and indirect economic costs of cancers

Table 3 illustrates direct and indirect costs of alcohol related cancers in Sri Lanka for 2015 which was USD 72.15 million. 36% (USD 25.67 million) of this cost consisted of direct costs while 64% (USD 46.47 million) consisted of indirect costs.

**Table 3:** Direct, indirect and total economic costs of alcohol related cancers in Sri Lanka 2105 in USD million.

Disease	Direct Costs				Indirect costs			Total
	In patient	Out patient	Out of Pocket	Total	Absen- teeism	Premature Mortality	Total	
**Breast**	0.22	0.05	0.32	**0.59**	0.49	0.28	**0.77**	**1.36**
**Colorectal**	0.68	0.04	0.42	**1.14**	0.77	0.74	**1.51**	**2.65**
**Larynx**	1.41	0.10	0.89	**2.40**	1.60	(a)	**1.60**	**4.00**
**Lip, oral cavity, pharynx**	9.03	0.46	6.03	**15.52**	11.74	14.90	**26.64**	**42.16**
**Liver**	0.47	0.00	0.15	**0.62**	0.35	0.66	**1.01**	**1.63**
**Oesophagus**	2.89	0.08	2.08	**5.05**	4.48	9.45	**13.93**	**18.98**
**Pancreas**	0.12	0.00	0.02	**0.14**	0.04	0.15	**0.19**	**0.33**
**Stomach**	0.14	0.00	0.08	**0.22**	0.17	0.66	**0.82**	**1.04**
**Total**	**14.96**	**0.73**	**9.99**	**25.68**	**19.64**	**26.84**	**46.47**	**72.16**

Note: The costs which were less that USD 0.05 million appear as zero. This is to ensure clarity and readability of the tables.

(a) Premature mortality costs of alcohol-related cancers of the Larynx was not calculated as the mortality was negligible.

The direct healthcare costs of alcohol-related cancers, which consist of the costs of inpatient care, outpatient care and OOPE were USD 25.67 million, which was 36% of the overall costs of alcohol-related cancers. The inpatient care costs contributed more than half (USD 14.96 million) of the direct cost. OOPE was USD 9.98 million, which was nearly 40% of the direct cost.

The indirect costs—the costs of absenteeism and premature mortality—consisted of 64% (USD 46.47 million) of the cost of alcohol-related cancers in 2015. The cost of premature mortality was USD 26.83 million, which was of 58% of the indirect cost. The cost of absenteeism was USD 19.64 million.

Overall, the costs of cancers of the upper aerodigestive tract (lip, oral cavity and pharynx and oesophagus) was UDS 61.14 million, which accounted for 85% of the total cost of alcohol related cancers.

### Direct and indirect economic costs of alcohol related noncommunicable diseases and other conditions

Table 4 contains the direct and indirect costs of alcohol related conditions other than cancers in Sri Lanka for 2015 which were taken into account for this study. These conditions consist of diseases conditions, different forms injuries, drowning and homicides. The total economic cost of conditions attributable to alcohol for 2015 was USD 814.16 million. 45% of this cost consisted of direct costs while 55% consisted of indirect costs.

**Table 4:** Direct, indirect and total economic costs of alcohol related conditions other than cancers in Sri Lanka 2105 in USD million.

	Direct Costs				Indirect costs			Total
Disease	In patient	Out patient	Out of Pocket	Total	Absen teeism	Premature Mortality	Total	
**Acute and chronic pancreatitis**	2.10	0.01	0.13	**2.23**	0.09	0.72	0.81	**3.04**
**Alcohol use disorders**	23.44	0.51	4.45	**28.40**	3.10	13.84	16.94	**45.34**
**Alcoholic Gastritis and Duodenitis**	39.58	2.77	14.64	**56.99**	10.81	-0.01	10.81	**67.80**
**Alcoholic liver disease**	15.20	0.19	1.49	**16.88**	1.05	77.70	78.75	**95.63**
**Cerebrovascular disease**	1.11	0.09	0.60	**1.79**	0.43	2.96	3.38	**5.18**
**Cholethaiasis and cholecystitis**	0.45	0.01	0.06	**0.51**	0.04	0.08	0.12	**0.63**
**Diabetes mellitus**	0.29	0.02	0.06	**0.37**	0.09	1.49	1.58	**1.95**
**Drowning**^**1**^	0.00	0.00	0.00	**0.00**	0.00	28.28	28.28	**28.28**
**Epilepsy**	0.05	0.05	0.23	**0.33**	0.17	8.81	8.98	**9.30**
**Falls**^**1**^	0.00	0.00	0.00	**0.00**	0.00	3.26	3.26	**3.26**
**Fire injuries/ burning**	0.31	0.00	0.15	**0.47**	0.10	2.78	2.88	**3.35**
**Homicide**^**1**^	0.00	0.00	0.00	**0.00**	0.00	2.40	2.40	**2.40**
**Hypertension**	0.56	0.09	0.37	**1.02**	0.28	2.07	2.35	**3.38**
**Ischemic Heart Disease**	28.71	0.17	1.60	**30.49**	1.11	9.11	10.22	**40.71**
**LRTI**	2.47	0.02	0.66	**3.14**	0.43	41.10	41.53	**44.67**
**Other diseases of liver**	0.07	0.04	0.26	**0.37**	0.19	8.51	8.69	**9.06**
**Poisoning**	7.59	0.04	0.88	**8.51**	0.58	1.61	2.19	**10.69**
**Road Injuries**	80.44	2.29	33.58	**116.31**	56.66	78.30	134.96	**251.28**
**Self-harm**	24.96	0.24	3.80	**29.00**	2.55	62.74	65.29	**94.29**
**Tuberculosis**	0.09	0.01	0.10	**0.20**	0.07	1.27	1.34	**1.54**
**Undiagnosed / Uncoded**	51.37	1.49	12.82	**65.67**	11.25	15.00	26.26	**91.93**
**Total**	**278.79**	**8.04**	**75.88**	**362.68**	**89.00**	**362.02**	**451.02**	**813.73**

^1^Number of hospital admissions were not available

The direct healthcare costs (the costs inpatient care, outpatient care and out of pocket expenditure) of these conditions amounted to USD 362.46 million. The inpatient care costs was UDS 278.79 million, which was more than 75% of the direct cost. The OOPE was USD 75.87 million and the outpatient care cost was USD 8.04 million.

The costs of absenteeism and premature mortality (indirect costs) of these alcohol related conditions were USD 451.02 million in 2015. The premature mortality cost was USD 362.42 million, which was 76% of the indirect cost. The cost of absenteeism was USD 89.00 million.

Road injury costs was the most significant contributor to the total economic costs of alcohol related conditions other than cancer. It was USD 251 million, which was 30.8% of the overall cost of these conditions. Alcoholic liver disease, alcoholic gastritis and duodenitis, self-harm, alcohol use disorders and alcohol associated lower respiratory tract infections were the other significant contributors to this cost.

The proportion of direct cost of ischemic heart disease and alcoholic gastritis and duodenitis were considerably high compared to their indirect cost component. This demonstrates the high economic burden imposed on the health care system by these conditions. The indirect cost of alcoholic liver disease was considerably high depicting the nature of high premature mortality with the condition.

### Total economic costs of alcohol

The total economic cost of alcohol in Sri Lanka was USD 885.85 million in 2015. USD 388.35 million (44%) consisted of direct costs, while USD 497.49 (66%) consisted of indirect costs. These costs are summarized in Table 5. The loss of productivity due to premature mortality, USD 388.86 million, was the highest cost category, accounting for 44% of the overall cost. The next highest cost was the inpatient care cost of USD 293.75 million, which was one third of the total cost.

**Table 5:** Summary of costing analysis in UDS million.

Cost category	Economic Cost
**Direct Cost**	
Inpatients care cost	**293.75**
Outpatient care cost	**8.77**
Out of Pocket expenditure	**85.87**
**Total**	**388.39**
**Indirect Cost**	
Cost of productivity loss due to Absenteeism	**108.64**
Cost of productivity loss due to premature mortality	**388.86**
**Total**	**497.50**
**Total Economic Cost**	**885.89**

## Discussion

As methodologies differ in studies calculating the direct and indirect costs of alcohol in different countries, comparison of cost components are technically not feasible. However, systematic reviews indicated that the indirect cost represent the largest cost of alcohol [13]. In Sri Lanka, the direct cost accounted for 44% of the total cost while indirect cost accounted for 56%.

The overall estimated present value of current and future economic cost of alcohol use (USD 885.89 million) was 1.07% of the GDP in Sri Lanka for 2015, which was USD 82,838.66 million (Central Bank of Sri Lanka, 2017). This figure is in line with the other countries where the cost of alcohol ranged from 0.45% to 5.44% of GDP and the direct cost accounted from 0.08% to 0.81% of GDP [13]. In this study, the direct cost accounted for 0.46% of the GDP of Sri Lanka in 2015.

When specific disease conditions are taken into account, the economic costs of the cancers of the lip, oral cavity, pharynx and oesophagus amounted to USD 61.14 million. It reflects the fact that Sri Lanka has one of the highest incidences of such cancers [23]—cancers of the lip, oral cavity and pharynx, are the commonest cancers among males in Sri Lanka with an incidence rate (ASR) of 19.4 per 100,000 population [22]. Alcohol related cancers of the liver and colon cost USD 1.63 and 2.65 million. Therefore, addressing alcohol use should be a major aspect of prevention of cancers in Sri Lanka. Road injuries accounted for USD 251.28 million, which was 28.5% of the total cost. Preventing such injuries need cooperation of many sectors other than health. This underlies the importance of multi-sectoral involvement in addressing alcohol related harm.

Spending on purchasing alcohol, absenteeism and OOPE due to alcohol related conditions can exacerbate and perpetuate poverty. The impact of alcohol on poverty occurs through many mechanisms and is seen even in high income counties [32] [33]. A study conducted in Sri Lanka examining the link between alcohol and poverty, 7% of men revealed that their alcohol expenditure was greater than their income [11]. Another study showed that the two lowest income categories spent more than 40% of their income on concurrent use of tobacco and alcohol [12]. Therefore, alcohol use and its consequences should be a major dimension in developing and implementing policies for alleviation of poverty in countries such as Sri Lanka. This study showed that OOPE accounted for 22% of the total direct cost (USD 85.87 million of USD 388.39 million), which would directly contribute to the overall effects on poverty.

Sri Lanka is a country providing free healthcare to all its citizens. People have the choice of seeking treatment in the government or the private sector for health services. The state sector is by far the largest provider of health services. The direct costs of in-patient care for alcohol related conditions, excluding out of pocket expenditure, (USD 293.75 million) was 39.7% of the recurrent health expenditure of the state health sector in 2015, which was USD 745.81 million [15]. This is a substantial cost, which underlies the importance and the priority required for effective initiatives to prevent the use and harm of alcohol.

The excise tax revenue from alcohol was USD 779.73 million in 2015 [15]. This was less than the estimated total economic cost of alcohol of USD 885.89 million, which as we have pointed out below, can be an underestimation due to methodological factors. Therefore, the short and medium-term alcohol taxation policies should take into account the revenue as well as cost aspects of alcohol use. Such policies therefore should have the objective of reducing harm from alcohol while optimizing revenue. In the longer-term, ideally, the country should aim not to be dependent on alcohol taxation as a significant source of revenue. In order to establish an effective alcohol taxation policy, appropriate econometric studies on alcohol related price elasticities, substitution effects etc. should be undertaken on a priority basis [34].

Studies estimating alcohol related harm in resource poor settings are sparse due to lack of data as well of paucity of technical expertise. The methodology used in this study can be used by countries in low resource settings where specific data is not available, to arrive at technically sound estimates of alcohol related harms. This, we feel, is one of the major contributions of this study to the literature quantifying harms of alcohol use.

## Conclusions

Alcohol related conditions imposed a significant economic burden to Sri Lanka in 2015, with indirect costs (66% of total) exceeding the direct costs (44%). Addressing alcohol use and its harms through effective implementation of evidence-based polices and interventions such as establishment of a taxation method that will continuously reduce affordability, strengthening enforcement of the current restrictions on advertising and promotions, enhancing the capacity and the priority given by the enforcement agencies to implement drink-driving counter measures and education of the public on different issues related to alcohol consumption is urgently required to address this economic burden.

## Limitations

The Centre for Disease Control and Prevention (CDC) identifies 54 acute and chronic conditions associated with alcohol [25]. We studied only 29 of these conditions due paucity of robust data of many conditions. Several types of costs were not included in the estimations. These include the opportunity costs of spending on purchasing alcohol products, costs of disease prevention and screening programmes, out of pocket expenditure by patients seeking services of the private sector, transport costs borne by the patient, enforcement and judicial costs and cost of property damage and insurance. Spending on drugs and devices which sometimes need to be purchased by the patients while obtaining treatment from the state sector was also not included in the estimate of out of pocket expenditure.

The economic cost of presenteeism (reduction in productivity despite working) due to alcohol related illness which has been taken into consideration in some studies [35] was also not calculated. Furthermore, intangible costs of the effects of alcohol were not included in the analysis. Studies show that intangible cost may account for 20% to 27% of the total cost of alcohol [13]. Recently, a study in Scotland found that the intangible cost accounted for the largest component of the total cost of alcohol (78%) [36]. The intangible costs in this study included costs of “pain, grief and suffering to the casualty, relatives and friends, and, for fatal casualties, the intrinsic loss of enjoyment of life, excepting consumption of goods and services”. The costs of alcohol related violence, suicides too were not included.

In estimating the costs of cancers, we calculated the attributable fractions using relative risks obtained from the systematic reviews, as relative risk data specific to Sri Lanka was not available. For conditions other than cancers, we used the country-specific estimates from the Global Burden of Disease (GBD) database [28] for most conditions. The GBD estimates were relatively conservative. Using conservative estimates for AFs for uncoded conditions too could have underestimated the actual alcohol related costs of these conditions.

In calculating indirect costs, we calculated the loss of income only up to the age of 70 years. In practical terms many in Sri Lanka earn a living beyond this age currently. Therefore, the overall economic cost estimated by this study is an underestimation of the actual cost.

## Supporting information

S1 Table Direct costs of cancer. (XLS)

S2 Table Indirect costs of cancer due to absenteeism. (XLS)

S3 Table Indirect costs of cancer due to premature mortality. (XLSX)

S4 Table Direct costs of NCD. (XLSX)

S5 Table Indirect costs of NCD due to absenteeism. (XLSX)

S6 Table Indirect costs of NCD due to premature mortality. (XLSX)
